# Does Temperature-Mediated Reproductive Success Drive the Direction of Species Displacement in Two Invasive Species of Leafminer Fly?

**DOI:** 10.1371/journal.pone.0098761

**Published:** 2014-06-06

**Authors:** Haihong Wang, Stuart R. Reitz, Juncheng Xiang, Guy Smagghe, Zhongren Lei

**Affiliations:** 1 State Key Laboratory for Biology of Plant Diseases and Insect Pests, Institute of Plant Protection, Chinese Academy of Agricultural Science, Beijing, People's Republic of China; 2 Department of Crop and Soil Science, Malheur County Extension, Oregon State University, Ontario, Oregon, United States of America; 3 Department of Crop Protection, Faculty of Bioscience Engineering, Ghent University, Ghent, Belgium; Natural Resources Canada, Canada

## Abstract

*Liriomyza sativae* and *L. trifolii* (Diptera: Agromyzidae) are two highly invasive species of leafmining flies, which have become established as pests of horticultural crops throughout the world. In certain regions where both species have been introduced, *L. sativae* has displaced *L. trifolii*, whereas the opposite has occurred in other regions. These opposing outcomes suggest that neither species is an inherently superior competitor. The regions where these displacements have been observed (southern China, Japan and western USA) are climatically different. We determined whether temperature differentially affects the reproductive success of these species and therefore if climatic differences could affect the outcome of interspecific interactions where these species are sympatric. The results of life table parameters indicate that both species can develop successfully at all tested temperatures (20, 25, 31, 33°C). *L. sativae* had consistently higher fecundities at all temperatures, but *L. trifolii* developed to reproductive age faster. Age-stage specific survival rates were higher for *L. sativae* at low temperatures, but these were higher for *L. trifolii* at higher temperatures. We then compared the net reproductive rates (*R*_0_) for both species in pure and mixed cultures maintained at the same four constant temperatures. Both species had significantly lower net reproductive rates in mixed species cultures compared with their respective pure species cultures, indicating that both species are subject to intense interspecific competition. Net reproductive rates were significantly greater for *L. sativae* than for *L. trifolii* in mixed species groups at the lower temperatures, whereas the opposite occurred at the higher temperature. Therefore, interactions between the species are temperature dependent and small differences could shift the competitive balance between the species. These temperature mediated effects may contribute to the current ongoing displacement of *L. sativae* by the more recent invader *L. trifolii* in warm climatic areas of China.

## Introduction

Species displacement, the elimination of a formerly established species from a habitat as a result of direct or indirect competitive interactions with another species, is a potentially widespread phenomenon [1], [2]. Evidence indicates that in most cases where two species interact in multiple environments, one species maintains its competitive superiority over the other [2]. An exception to this pattern has been observed in interactions between two leafminer species in the genus *Liriomyza* (Diptera: Agromyzidae). *L. sativae* Blanchard and *L. trifolii* (Burgess) have both displaced and been displaced by the other in different locations [2], [3].

These closely related species have similar life histories [4]. Females oviposit directly into foliar tissue, and the larvae forage by mining through the palisade mesophyll of leaves. Mature larvae cut an exit hole, from which they crawl out and drop to the ground to pupate. Both species are highly polyphagous and can be severe pests of numerous horticultural crops [5].

The two species are endemic to the Americas [6], but they have rapidly spread throughout the world through human mediated transport [4]. In China, *L. sativae* was first found in October 1993 on Hainan Island, and spread northwardly throughout China within a few years [7], [8]. *L. trifolii* was first recorded in Guangdong province and Hainan Island in 2005 and 2006, respectively [9]. Since then *L. sativae* has been displaced by *L. trifolii* on Hainan Island, but it has persisted as an important pest in inland areas of China. In the USA, *L. trifolii* rapidly replaced *L. sativae* as the predominant leafminer pest of vegetables and ornamentals in the Mediterranean climates of California and in other regions of the western USA, following its introduction from Florida in the 1970s [10], [11]. In Japan, *L. trifolii* and *L. sativae* were first found in 1990 and 1999, respectively [12], [13], and each species rapidly expanded its range soon after their introductions [14]. In Kyoto Prefecture, *L. trifolii* and *L. sativae* were found to coexist on the same host plants in 1999. However since 2000, *L. sativae* has become the dominant species, while *L. trifolii* is now rarely found [15].

Where the displacement of one of these species by the other has been studied, no unique mechanisms have been identified as causing the displacements. The main reason for the displacement of *L. sativae* by *L. trifolii* in Hainan Island, China and California, USA, has been thought to be the lower susceptibility of *L. trifolii* populations to many commonly used insecticides [2], [3]. In contrast, Abe and Tokumaru [13] concluded that the reason for the displacement of *L. trifolii* by *L. sativae* in Kyoto Prefecture of Japan is the higher fecundity of *L. sativae* and differential effects of the local parasitoid complex on the two *Liriomyza* species. These mechanisms are likely to be mediated by other local environmental conditions, including temperature.

Temperature is one of the most important factors affecting the distribution and abundance of poikilothermic animals, including insects [16], [17]. The latitudinal and altitudinal distributions of *Liriomyza* species are directly dependent on environmental temperature [18], [19]. When reared individually, females*L. Sativae* tend to produce more eggs than do females of *L. trifolii* throughout the temperature ranges [20]. However when coexisting in nature, *L. trifolii* tends to outperform *L. sativae* in higher-temperature regions, such as in Hainan, China and California, USA, where as *L. sativae* tends to outperform *L. trifolii* in lower-temperature regions, such as in Kyoto, Japan [13]. Reports from other organisms, such as fishes, show that temperature drives differential competitive interaction among species [21]–[23], and temperature also differentially affects the fitness of insecticide resistant and susceptible aphids, *Myzus persicae*, and house flies, *Musca domestica* [24].

Mean air temperatures during the growing season in agricultural areas of Hainan, China and California, USA, tend to be higher than in Kyoto, Japan. For example, the average daily mean and maximum temperaturesare greater for 11 months of the year in Sanya, Hainan Island, China and in Fresno, California, USA than in Kyoto, Japan (Western Regional Climate Center, http://www.wrcc.dri.edu/; China Meteorological Administration, http://www.cma.gov.cn/; and World Weather Online, http://www.worldweatheronline.com/). Mean daily maximum temperatures per month are up to 17°C greater in Sanya than in Kyoto, and up to 6°C great in Fresno than in Kyoto. Consequently, differential effects of temperature on the reproductive success and competitive interactions of these species may contribute to displacements occurring in different directions in these localities.

To determine if temperature-mediated variation in reproductive success could account for species displacements occurring in different directions, we conducted a series of studies comparing the reproductive success and life table parameters of *L. sativae* and *L. trifolii* under different temperature regimes.

## Materials and Methods

### Ethics statement

No specific permissions were required for these locations or activities. The location is not privately-owned or protected in any way. The field studies did not involve endangered or protected species.

### Insect Strains

Populations of both *L. sativae* and *L. trifolii* were collected and maintained according to previously described methods [3]. Briefly, individuals of both species were collected from a cowpea, *Vigna unguiculata* L. Walp., field in Sanya, Hainan Province (18.25°N, 109.50°E), in 2007. Both populations were subsequently reared separately on cowpea plants at the Sanya Experiment Station under controlled conditions (25±1°C, 70±10% relative humidity [RH], 16∶8 light∶dark [L∶D] photoperiod). Additional wild collected flies were added regularly to these colonies to reduce potential effects of inbreeding. Thirty individuals from each population were identified to species by microscopic observation and/or mitochondrial sequence analysis [25], [26] before initiation of the experiments in 2012, and the results showed that the colonies only contained individuals of the appropriate species.

### Population dynamics in response to temperature

To assess the potential of *L. sativae* and *L. trifolii* populations to increase under low density at different temperatures, the demographics of each species were determined on cowpea plants, according to the method described by Lanzoni et al. [27]. We selected four experimental temperatures (20, 25, 31, 33°C) that are within the range of temperatures at which each species can develop and at which they are routinely exposed to in the environment [20]. For each temperature and each species, ten plastic pots (11 cm in top diameter, 9 cm in bottom diameter, and 13 cm in height) each containing four bean plants (first true leaves fully expanded) were exposed to leafminer adults for 3–5 h. To minimize adverse effects of intraspecific competition among larvae [28], [29], exposure time was regulated according to the number of adults (shorter exposure time if flies density was high and vice versa) to limit the number of larvae ≤8 per leaf. After exposure, bean plants were checked for the presence of flies, and transferred to environmental chambers at constant temperatures of 20, 25, 31 or 33±1°C, 75±15% RH, and a photoperiod of 16∶8 (L∶D). Progress in immature development and mortality of egg and larval stages were assessedevery 8 h (0600, 1400 and 2200 hours). Eggs were located using transmitted light at 25–50× magnification. If the number of eggs within a leaf exceeded 8, the leaf was not used in the experiment. The initial number of eggs used in each temperature ranged from 85 to 120 and 90 to 118 per replicate for *L. sativae* and *L. trifolii*, respectively.

The period during which a larva was outside the leaf before pupariation was included as part of the pupal development time. After pupariation, offspring were transferred individually to small glass scintillation vials, and checked daily for adult eclosion. These individuals were held at their respective larval rearing temperatures.

Newly emerged adults were sexed and subsequently paired in rearing containers with bean plants and maintained at their respective larval rearing temperatures. Males from rearing colonies were added in cases where the number of males was less than the number of females, or if males in the mating pairs died before the female. Bean plants were replaced with new ones daily. Longevity of both sexes and female fecundity, as determined by the number of eggs laid, were checked daily.

Data on survivorship, longevity, and female daily fecundity of *L. sativae* and *L. trifolii* were analyzed according to age-stage, two-sex life tables [30]–[32], using the computer program TWOSEX-MSChart [33]. Total preovipositionperiod (TPOP) was calculated as the duration between the birth and the first oviposition day for each individual female. The population parameters calculated were intrinsic rate of increase (*r*), gross reproductive rate (*GRR*), net reproductive rate (*R_0_*) and the mean generation time (*T*). The intrinsic rate of increase was estimated by using the iterative bisection method and the Euler-Lotka equation:
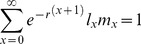
(1)with the age indexed from 0[31], in which *l_x_* is the age-specific survival rate and *m_x_* is the age-specific fecundity of the total population. The mean generation time was defined as the length of time that a population would need to increase to *R_0_*-fold of its size (i.e., *e^rT^* = *R_0_*) at the stable age-stage distribution. The mean generation time was calculated as *T* = (ln*R_0_*)/*r*. Before analysis, development time and fecundity were square root transformed. As the assumptionsof normal distribution for parametric analysis were not fulfilled, the data were analysed using Mann-Whitney tests (*U* tests) (Sigmaplot12.0, Systat Software Inc., Chicago, IL) to evaluate the differences between the two leafminer species. A bootstrap technique [34] was used to estimate the means and standard errors of population parameters. The differences in the population parameters were compared by using the Tukey-Kramer technique.

### Comparison of reproductive success in pure and mixed species cultures

To assess interspecific competition between *L. trifolii* and *L. sativae* under different temperatures, a series of laboratory experiments was undertaken, with methods similar to those used by Tantowijoyo and Hoffmann [19]. Temperature settings for the experiments were 20, 25, 31, and 33°C, with ±0.5°C fluctuation. At each temperature, three experimental treatments were established: *L. trifolii* and *L. sativae* in mixed species cultures, *L. sativae* in pure culture, and *L. trifolii* in pure culture. For each experimental replicate, twenty newly-emerged (<24 h) adults were released into a mesh cage. In the mixed species cultures, there was a 1∶1 ratio of *L. sativae* to *L. trifolii*. Equal numbers of females and males were used for each species in all replicates. Cages were 30×50×47 cm and held one pot containing four bean plants. Bean plants were 3–4 weeks post-sowing and had two fully-developed true leaves and three cotyledons. Experiments for both species under each temperature were run simultaneously. There were three replicate cages for each temperature and species composition treatment.

Every 1 day (for 31 and 33°C), 2 days (for 25°C), or 4 days (for 20°C), bean plants were replaced by new ones. By doing so, comparable numbers of larvae per leaf (range 19 to 28 individuals) were achieved across the different temperature conditions. Infested plants were kept in separate boxes without adult flies.

When it became apparent that larvae were ready to exit their mines, infested leaves were harvested and stored in plastic containers to collect puparia. Puparia were collected and placed into plastic vials for adult eclosion. Plants and insects were maintained at their respective experimental temperature throughout the trial. Adults were counted upon eclosion and identified to species based on microscopic observation and/or mitochondrial sequence analysis [6], [35].

Comparisons of net reproductive rates (*R*_0_) were used to assess competitive performance between the two leafminer species [20], [36]. Briefly, *R*_0_ was defined as the number of offspring produced by a leafminer species divided by the initial number of that species. *R*_0_ values were log-transformed (*lnR*_0_) before analysis. Analysis of variance (ANOVA) was used to determine if reproductive success was influenced by species, culture type (single or mixed species), temperature, and the interactions of these factors. The full model included the fixed effects of species (1 df), culture type (pure or mixed species; 1 df), temperature treatment (3 df) and their interactions. To determine responses to temperature within each species and culture type, the temperature effect was partitioned into single-degree-of freedom contrasts to test for linear and quadratic trendsin the response of reproductive success [37].

Intraspecific competition has been observed in both species [38], [39]. If interspecific competition for a species was more intense than intraspecific competition for that species, we would expect that its *R*_0_ in mixed culture to be lower than the *R*_0_ in pure culture. Interspecific differences in *R*_0_ in mixed populations would indicate where one species has a competitive advantage over the other. If the intensity of interspecific competition varied with temperature, then temperature could mediate the outcome of interspecific interactions.

## Results

### Comparison of population dynamics in response to temperature

The developmental periods for each stage, adult longevity, preoviposition period, and female fecundity of the two leafminer species under different temperatures are given in Table 1. Significant interspecific differences in some of these life history traits were observed. Where there were differences between the species at the same temperature, rates were faster for *L. trifolii* than for *L. sativae*. Most importantly from a population dynamics perspective, the mean total preoviposition period (TPOP), which is the age at which a female begins oviposition, was significantly less for *L. trifolii* than for *L. sativae* at all temperatures (Table 1). This difference would allow *L. trifolii* females to begin reproducing at an earlier point in their life than *L. sativae* could.

**Table 1:** Developmental duration of various life stages and female fecundity of *L. trifolii* and *L. sativae* when reared at different constant temperatures.

Statistic	Species	Temperature (°C)			
		20	25	31	33
Egg stage (d)	*L. sativae*	4.77±0.05 b##	2.30±0.04 a	1.89±0.03 b	1.76±0.05 b
	*L. trifolii*	4.64±0.05 a	2.21±0.04 a	1.52±0.05 a	1.58±0.05 a
Larval stage (d)	*L. sativae*	8.79±0.06 b	4.64±0.07 a	3.57±0.06 a	3.29±0.06 a
	*L. trifolii*	8.39±0.92 a	4.45±0.06 a	3.63±0.07 a	3.06±0.03 a
Pupal stage (d)	*L. sativae*	14.58±0.05 a	9.49±0.05 a	7.63±0.09 a	7.25±0.13 a
	*L. trifolii*	14.39±0.09 a	9.23±0.07 a	7.42±0.09 a	6.91±0.06 a
Total preoviposition period (TPOP) (d)#	*L. sativae*	28.16±0.12 b	16.36±0.15 b	13.05±0.14 b	12.5±0.23 b
	*L. trifolii*	27.46±0.21 a	15.83±0.11 a	12.51±0.13 a	11.35±0.09 a
Fecundity (eggs/female)	*L. sativae*	77.67±1.85 b	146.70±3.71 b	111.68±3.82 b	72.86±2.79 b
	*L. trifolii*	52.37±1.47 a	108.52±3.11 a	101.37±2.90 a	68.14±2.59 a

# TPOP, total preoviposition period, the duration from birth to first reproduction.

## Means within each statistical category and temperature followed by the same letters are not significantly between the two species (P>0.05).

The mean fecundity for individual females also varied with temperature (Table 1), but regardless of temperature, the mean number of eggs laid by *L. sativae* females was greater than that for *L. trifolii* females. However, the differences in fecundity between the species became less pronounced with increasing temperature. For example, at 20°C, *L. sativae* females laid >48% more eggs than did *L. trifolii* females, but the difference was <7% at 33°C.

**Figure 1:**
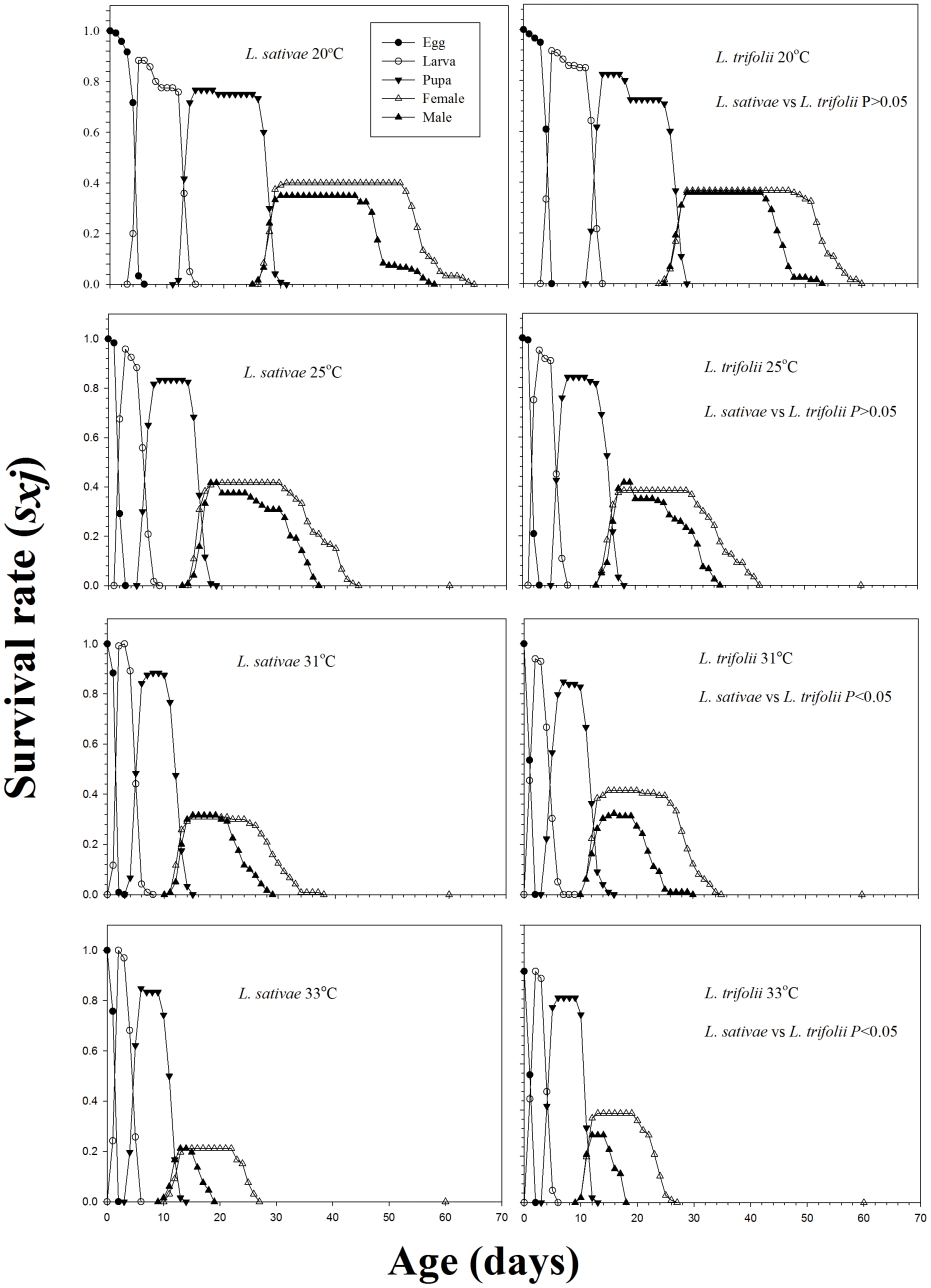
Age-stage specific survival rate (*s_xj_*) of *Liriomyza sativae* and *L. trifolii* under different constant temperatures.

The curves for the age-stage survival rate (*s_xj_*) show the probability that an egg will survive to age *x* and stage *j* (Fig. 1). At lower temperatures, the probabilities that a newly laid egg of *L. trifolii* would survive to the adult stage (0.73 and 0.80 at 20, and 25°C, respectively) were comparable with those of *L. sativae* (0.75 (Mann-Whitney tests (*U* tests) *P*>0.05) and 0.83 (Mann-Whitney tests (*U* tests) *P*>0.05) at 20 and 25°C, respectively). However, at higher temperatures, the probabilities that *L. trifolii* progeny would survive to the adult stage (0.75 and 0.73 at 31 and 33°C, respectively) were significantly higher than those for *L. sativae* (0.67 (Mann-Whitney tests (*U* tests) *P*<0.05) and 0.48 (Mann-Whitney tests (*U* tests) *P*<0.05) at 31 and 33°C, respectively).

These biological differences were subsequently expressed in the differences in the intrinsic rate of increase and other life table parameters between the species (Table 2). Because of its faster development time to reproductive age, *L. trifolii* had a significantly shorter generation time than *L. sativae* at all temperatures. At lower temperatures (20 and 25°C), the intrinsic rate of increase (*r*) was greater for *L. sativae* than for *L. trifolii*. However, at the higher temperatures (31 and 33°C), these parameters were greater for *L. trifolii* than for *L. sativae*. These differences resulted primarily from the greater preadult mortality of *L. sativae* at higher temperatures (Fig. 1) and the longer time it took *L. sativae* to begin reproduction (Table 1). Greater fecundity coupled with similar survival rates meant that at lower temperatures (20 and 25°C), the gross reproductive rates (*GRR*) and the net reproductive rates (*R_0_*) for *L. sativae* were higher than those for *L. trifolii* (Table 2). However, at higher temperature (31 and 33°C), these two reproductive rates were lower for *L. sativae* than for *L. trifolii*. In terms of numbers of eggs laid, the fecundity of *L. sativae* was higher than that of *L. trifolii* (Table 1). However, its survival rates declined with temperature, whereas survival rates of *L. trifolii* were relatively constant across the temperature range (Figure 1).This difference would allow *L. trifolii* to have greater reproductive success at higher temperatures despite laying fewer eggs.

**Table 2:** Estimates of life table population parameters (*x* ± SE) for *Liriomyza sativae* and *L. trifolii* reared under different constant temperatures.

Population parameter	Species	Temperature (°C)			
		20	25	31	33
*r*(d^−1^)	*L. sativae*	0.096±0.004 a#	0.186±0.005 a	0.194±0.082 b	0.171±0.01 b
	*L. trifolii*	0.095±0.004 b	0.178±0.006 b	0.224±0.008 a	0.229±0.010 a
GRR	*L. sativae*	41.65±4.23 a	77.28±7.74 a	61.63±6.74 a	43.51±6.80 a
	*L. trifolii*	30.82±2.91 b	55.55±5.88 b	58.51±6.12 b	40.38±4.36 b
*R_0_*	*L. sativae*	31.17±3.58 a	61.10±6.80 a	34.56±4.83 b	15.41±3.69 b
	*L. trifolii*	21.82±2.93 b	41.62±4.95 b	41.95±5.17 a	26.24±3.49 a
*T*	*L. sativae*	35.68±0.19 a	22.10±0.17 a	18.18±0.20 a	15.86±0.25 a
	*L. trifolii*	32.13±0.17 b	20.19±0.19 b	16.63±0.18 b	14.23±0.12 b

# Means of parameter estimates within each temperature with the same lowercase letters are not significantly different (P>0.05).

### Comparison of the potential for interspecific competition between two leafminer species under different temperatures

The net reproductive rates for pure cultures of *L. sativae* showed a pronounced curvilinear response with temperature (Table 3; contrast for linear effect of temperature on *R_0_*: *F = *24.63; df = 1, 32; P<0.0001; contrast for quadratic effect: *F = *74.40; df = 1, 32; P<0.0001). Reproductive success for *L. sativae* peaked at 25and 31°C; its reproductive success was significantly lower at the extreme lower and upper temperatures of 20 and 33°C than at 25 or 31°C (Table 4; P<0.005). There was no significant difference in net reproductive rates at these two extreme temperatures (P = 0.35).

**Table 3:** Analysis of variance (ANOVA) results for the net reproductive rate (*R_0_*) of *Liriomyza sativae* and *L. trifolii* in pure and mixed cultures maintained at four different temperatures#.

Source	df	F	P
Species	1	13.80	0.0008
Culture Type	1	265.85	<0.0001
Temperature	3	89.64	<0.0001
Species×Culture Type	1	0.13	0.72
Species×Temperature	3	32.24	<0.0001
Culture Type×Temperature	3	0.47	0.71
Species×Culture Type×Temperature	3	5.00	0.006
Error	32		

# Values for *R_0_* were logarithmically transformed [ln(*R_0_*)] before analysis.

**Table 4:** Net reproductive rates (*R_0_*) for *Liriomyza sativae* and *L. trifolii* in pure and mixed cultures maintained at four different temperatures#.

Temperature (°C)	Pure culture	Mixed culture		Pure culture
	*L. sativae*	*L. sativae*	*L. trifolii*	*L. trifolii*
20	33.17 (26.61–41.34) A##b###	17.14 (13.75–21.37) Bc	5.19 (4.17–6.47) Be	14.91 (11.96–18.59) Ac
25	70.54 (56.59–87.93) Aa	35.49 (28.47–44.23) Ba	22.36 (17.94–27.86) Bbc	59.51 (47.75–74.18) Aa
31	53.77 (43.15–67.03) Aa	21.08 (16.91–26.27) Bbc	24.76 (19.87–30.86) Bb	52.54 (42.15–65.48) Aa
33	28.69 (23.02–35.77) Aa	7.66 (6.15–9.55) Bd	16.54 (13.27–20.61) Bc	32.09 (25.75–40.00) Ab

# Values for *R_0_* were logarithmically transformed [ln (*R_0_*)] before analysis. Back transformed means and their 95% confidence limits (in brackets) are presented.

## For each species, means within rows followed by the same uppercase letter are not significantly different (P>0.05).

### For each culture type, means followed by the same lower case letter are not significantly different (P>0.05).

Reproductive success of pure cultures of *L. trifolii* also showed a curvilinear increase with temperature; there was a consistent linear increase in *R*_0_ with increasing temperature in addition to the significant quadratic effect of temperature (Table 3; contrast for linear effect of temperature on *R*_0_ *: F = *69.59; df = 1, 32; P<0.0001; contrast for quadratic effect: *F = *62.42; df = 1, 32; P<0.0001). As with *L. sativae*, the net reproductive rate for *L. trifolii* also peaked at 25–31°C (Table 4).

Net reproductive rates for both species in mixed cultures also showed curvilinear relationships with temperature (Tables 3 and 4; *L. sativae* contrast for linear effect of temperature on *R*_0_: *F = *0.42; df = 1, 32; P = 0.52; contrast for quadratic effect: *F = *43.24; df = 1, 32; P<0.0001; *L. trifolii* contrast for linear effect of temperature on *R*_0_: *F = *29.55; df = 1, 32; P<0.0001; contrast for quadratic effect: *F = *71.54; df = 1, 32; P<0.0001). When in mixed cultures both *L. sativae* and *L. trifolii* had lower net reproductive rates than when in pure cultures; this difference occurred at all of the tested temperatures (Table 4). These results indicate that both species suffered from interspecific competition and that interspecific competition was more intense than was any intraspecific competition that may have been present.

Although both species were subject to interspecific competition, the pattern of the effect changed with temperature. At the lower temperatures of 20 and 25°C, *L. sativae* had significantly higher net reproductive rates in mixed cultures than did *L. trifolii*. At 31°C, there was no significant difference between the species, but at 33°C, *L. trifolii* had a significantly higher R_0_ than *L. sativae*.

The relative difference in net reproductive rates between mixed and pure cultures for each species also was temperature dependent (Table 4). At the lower temperatures, net reproductive rates for *L. sativae* in mixed cultures were approximately one half of the corresponding rates for pure cultures. At 31 and 33°C, the net reproductive rates for *L. sativae* in mixed cultures were only 27–40% of the corresponding rates in pure cultures. However, *L. trifolii* showed an opposite response. At the higher temperatures, its net reproductive rates in mixed species cultures were approximately half of its corresponding pure culture rates. At the lower temperatures, the effect of *L. sativae* was much greater, with the net reproductive rates for *L. trifolii* in mixed species cultures being 35–38% of the rates for *L. trifolii* in pure cultures.

## Discussion

*Liriomyza sativae* and *L. trifolii* have become cosmopolitan pest species through human mediated movement over the past 40 years [4]–[6]. Both species have successfully colonized numerous novel habitats with differing climatic and environmental conditions. The two species have also come into contact with each other in these new locations, which has led to cases of displacement occurring. These cases have been bidirectional with the initial colonizing species being displaced by the subsequent invading species. Because of the historic nature of species displacements, cases where a species fails to become established because of the presence of a superior competitor (i.e., the second invader is repulsed) are not likely to be evident.

Our results show that both *L. sativae* and *L. trifolii* can reproduce successfully over a similar range of temperatures. In isolation, both species had net reproductive rates far greater than 1 and consequently, their intrinsic rates of increase were greater than 0 at all temperatures. These results indicate that both species would be able to successfully colonize the same habitats and that in isolation their populations would increase and become established.

However, our results demonstrate that the reproductive success of both *L. sativae* and *L. trifolii* is severely constrained by the presence of the other species, suggesting that a significant degree of interspecific competition can occur between these species. Although our study was not designed to determine specific mechanisms of interspecific competition, previous studies have identified different forms of intraspecific competition that can occur within these species. Because of the similar life histories of these species, interspecific competition may be expected to operate in a similar manner as intraspecific competition. Larvae are generally confined to their natal leaves, which are spatially isolated from each other. Therefore, intraspecific and/or interspecific competition is likely to occur among larvae within a particular leaf. Interference competition occurs among first and second instars when they may cannibalize one another [39]. Although third instars are not cannibalistic, they consume ∼80% of the total leaf tissue consumed during the entire larval stage [40]. Therefore, this stage would be most responsible for exploitative competition resulting from the depletion of host leaf resources. Size and survival to the adult stage decrease significantly as resources are depleted [38], [39].

*L. trifolii* would be expected to have a competitive advantage over *L. sativae* in these types of interactions because of its faster development through the egg and larval stages at most temperatures. This advantage would apply to both interference and exploitative competition. Faster development through the egg and larval stages at most temperatures would allow *L. trifolii* larvae to escape attacks by *L. sativae* larvae. In addition, its larvae could obtain necessary resources more rapidly than *L. sativae*, and at high densities, *L. sativae* may not be able to acquire sufficient resources to complete development. Such advantages of *L. trifolii* could increase over multiple generations given the greater speed with which females reach reproductive age. These factors may allow it to outcompete *L. sativae*, especially at higher temperatures where it had higher survivorship than *L. sativae* and differences in fecundity are less pronounced. However, these advantages would be counteracted by the greater fecundity and probability of survivorship of *L. sativae* at lower temperatures, which would change the competitive advantage in favor of *L. sativae* under those conditions.

Our results provide evidence that significant differences in interspecific interactions can occur within a relatively narrow temperature range. Slight differences in temperature are known to alter the outcome of competitive interactions of species with similar life histories to *Liriomyza* spp [25], [41]. Other factors, such as differential effects of natural enemies, host plants or insecticides also are likely to influence the populations of these pests, but it is clear that temperature can be a significant factor in altering competitive interactions between these species. Changes in population dynamics and demographics are likely to take place within a growing season as seasonal weather conditions change, but these may also translate over time into long term shifts in the demographics within a region, and ultimately drive the displacement of one species of *Liriomyza* by another. More favorable climatic conditions therefore may be facilitating the ongoing displacement of *L. sativae* by *L. trifolii* in southern China.
